# Supplementary material for: The Association between Proton Pump Inhibitors and the Effectiveness of CDK Inhibitors in HR+/HER- Advanced Breast Cancer Patients: A Systematic Review and Meta-Analysis
Source: Cancers (Basel). 2023 Oct 25;15(21):5133. doi: 10.3390/cancers15215133 (PMC10649865; doi:10.3390/cancers15215133)
Supplement: Supplementary file 1 [file cancers-15-05133-s001.zip › cancers-2662274-supplementary.pdf]

Supplementary file

The following material supplements the article:

" The Association between Proton Pump Inhibitors and the Effectiveness of CDK Inhibitors in HR+/HER- Advanced Breast Cancer Patients: A Systematic Review and Meta-Analysis"

Contents of supplements

Table S1. Preferred Reporting Items for Systematic Reviews and Meta-Analyses (PRISMA) 2020 checklist

Table S2. Data synthesis

Figure S1. Visual summary of ROBINS-I for survival outcomes

Figure S2. Leave-one-out analysis of comparative overall survival and progression-free survival between PPI users and non-users in HR+/HER2- metastatic breast cancer patients taking Palbociclib.

Figure S3. Forest plot of adjusted survival outcomes between PPI users and nonusers in patients taking Palbociclib

Figure S4. Forest plot of unadjusted survival outcomes between PPI users and nonusers in patients taking Palbociclib after the exclusion of studies subject to serious risk of bias

Figure S5. Leave-one-out analysis of comparative progression-free survival between PPI users and non-users in HR+/HER2- metastatic breast cancer patients taking Ribociclib.

Figure S6. Leave-one-out analysis of risk ratio of CDKi dose reduction due to drug toxicity between PPI users and non-users in HR+/HER2- metastatic breast cancer patients taking Palbociclib and Robociclib

This supplementary material has been provided by the authors to give readers additional information about their work

Table S1. PRISMA 2020 checklist.

Section and Topic	Item #	Checklist item	Location where item is reported
**TITLE**			
Title	1	Identify the report as a systematic review.	Page 1
**ABSTRACT**			
Abstract	2	See the PRISMA 2020 for Abstracts checklist.	Page 2
**INTRODUCTION**			
Rationale	3	Describe the rationale for the review in the context of existing knowledge.	Page 3
Objectives	4	Provide an explicit statement of the objective(s) or question(s) the review addresses.	Page 3
**METHODS**			
Eligibility criteria	5	Specify the inclusion and exclusion criteria for the review and how studies were grouped for the syntheses.	Page 4
Information sources	6	Specify all databases, registers, websites, organisations, reference lists and other sources searched or consulted to identify studies. Specify the date when each source was last searched or consulted.	Page 4
Search strategy	7	Present the full search strategies for all databases, registers and websites, including any filters and limits used.	eMethod 2
Selection process	8	Specify the methods used to decide whether a study met the inclusion criteria of the review, including how many reviewers screened each record and each report retrieved, whether they worked independently, and if applicable, details of automation tools used in the process.	Page 4

Section and Topic	Item #	Checklist item	Location where item is reported
Data collection process	9	Specify the methods used to collect data from reports, including how many reviewers collected data from each report, whether they worked independently, any processes for obtaining or confirming data from study investigators, and if applicable, details of automation tools used in the process.	Page 4
Data items	10a	List and define all outcomes for which data were sought. Specify whether all results that were compatible with each outcome domain in each study were sought (e.g. for all measures, time points, analyses), and if not, the methods used to decide which results to collect.	Page 4
	10b	List and define all other variables for which data were sought (e.g. participant and intervention characteristics, funding sources). Describe any assumptions made about any missing or unclear information.	Page 4
Study risk of bias assessment	11	Specify the methods used to assess risk of bias in the included studies, including details of the tool(s) used, how many reviewers assessed each study and whether they worked independently, and if applicable, details of automation tools used in the process.	Page 4
Effect measures	12	Specify for each outcome the effect measure(s) (e.g. risk ratio, mean difference) used in the synthesis or presentation of results.	Page 4
Synthesis methods	13a	Describe the processes used to decide which studies were eligible for each synthesis (e.g. tabulating the study intervention characteristics and comparing against the planned groups for each synthesis (item #5)).	Page 4
	13b	Describe any methods required to prepare the data for presentation or synthesis, such as handling of missing summary statistics, or data conversions.	Page 4
	13c	Describe any methods used to tabulate or visually display results of individual studies and syntheses.	eMethod 3
	13d	Describe any methods used to synthesize results and provide a rationale for the choice(s). If meta-analysis was performed, describe the model(s), method(s) to identify the presence and extent of statistical heterogeneity, and software package(s) used.	eMethod 3

Section and Topic	Item #	Checklist item	Location where item is reported
	13e	Describe any methods used to explore possible causes of heterogeneity among study results (e.g. subgroup analysis, meta-regression).	Page 4
	13f	Describe any sensitivity analyses conducted to assess robustness of the synthesized results.	Page 4
Reporting bias assessment	14	Describe any methods used to assess risk of bias due to missing results in a synthesis (arising from reporting biases).	N/A
Certainty assessment	15	Describe any methods used to assess certainty (or confidence) in the body of evidence for an outcome.	N/A
**RESULTS**			
Study selection	16a	Describe the results of the search and selection process, from the number of records identified in the search to the number of studies included in the review, ideally using a flow diagram.	Page 5
	16b	Cite studies that might appear to meet the inclusion criteria, but which were excluded, and explain why they were excluded.	Figure 1
Study characteristics	17	Cite each included study and present its characteristics.	Page 5
Risk of bias in studies	18	Present assessments of risk of bias for each included study.	Page 5
Results of individual studies	19	For all outcomes, present, for each study: (a) summary statistics for each group (where appropriate) and (b) an effect estimate and its precision (e.g. confidence/credible interval), ideally using structured tables or plots.	Page 5
Results of	20a	For each synthesis, briefly summarise the characteristics and risk of bias among contributing studies.	Page 5

Section and Topic	Item #	Checklist item	Location where item is reported
syntheses	20b	Present results of all statistical syntheses conducted. If meta-analysis was done, present for each the summary estimate and its precision (e.g. confidence/credible interval) and measures of statistical heterogeneity. If comparing groups, describe the direction of the effect.	Page 6
	20c	Present results of all investigations of possible causes of heterogeneity among study results.	Page 5
	20d	Present results of all sensitivity analyses conducted to assess the robustness of the synthesized results.	Page 5
Reporting biases	21	Present assessments of risk of bias due to missing results (arising from reporting biases) for each synthesis assessed.	N/A
Certainty of evidence	22	Present assessments of certainty (or confidence) in the body of evidence for each outcome assessed.	N/A
**DISCUSSION**			
Discussion	23a	Provide a general interpretation of the results in the context of other evidence.	Page 6
	23b	Discuss any limitations of the evidence included in the review.	Page 6
	23c	Discuss any limitations of the review processes used.	Page 6
	23d	Discuss implications of the results for practice, policy, and future research.	Page 6
**OTHER INFORMATION**			
Registration and protocol	24a	Provide registration information for the review, including register name and registration number, or state that the review was not registered.	Page 3
	24b	Indicate where the review protocol can be accessed, or state that a protocol was not prepared.	Page 3
	24c	Describe and explain any amendments to information provided at registration or in the protocol.	Page 3

Section and Topic	Item #	Checklist item	Location where item is reported
Support	25	Describe sources of financial or non-financial support for the review, and the role of the funders or sponsors in the review.	N/A
Competing interests	26	Declare any competing interests of review authors.	N/A
Availability of data, code and other materials	27	Report which of the following are publicly available and where they can be found: template data collection forms; data extracted from included studies; data used for all analyses; analytic code; any other materials used in the review.	N/A

Table S2. Data synthesis

We used Rstudio with meta, metafor, and netmeta packages to conduct statistical analysis:

【meta】package
Random-effects model:
Meta-analysis of binary outcome data (metabin)
Meta-analysis of continuous outcome data (metacont)
Generic inverse variance meta-analysis (metagen)
Several plots for meta-analysis: Forest plot (forest)
Leave-one-out analysis: (metainf)

```
#Meta-analysis

library(meta)
library(xlsx)

cdk1<-read_xlsx('/Users/kychi/Desktop/all/ICI-series study/CDK PPI/CDK.xlsx',
sheet = 'Palbociclib')
cdk1survival<-metagen(TE, seTE, studlab = study, data = cdk2.2, comb.fixed = F,
                    sm = 'HR', method.tau = 'REML', byvar = outcome)

forest(cdk_OS, layout = 'RevMan5', lab.e="With PPI", lab.c="Without PPI",
        xlab="Favors PPI users                    Favors PPI non-users",
ff.xlab="bold", overall = F, overall.hetstat = F,vcol.by="black", comb.fixed=F,
col.diamond.random='black', col.diamond.lines.random='black',test.subgroup =
F,vcol.square = 'blue', col.square.lines = 'blue', print.byvar = F, test.subgroup.random
= F,vfontsize = 8, spacing = 0.8)

cdk2<-read_xlsx('/Users/kychi/Desktop/all/ICI-series study/CDK PPI/CDK.xlsx',
sheet = 'AE')
cdk_ae<-metabin(event.e, n.e, event.c, n.c, studlab = study, data = cdk3, sm = 'RR',
method.tau = 'REML', byvar = regimen, overall = F, overall.hetstat = F)
forest(cdk_ae, layout = 'RevMan5', lab.e="With PPI", lab.c="Without PPI",
        xlab="Favors PPI users                    Favors PPI non-users",
ff.xlab="bold", overall = F, overall.hetstat = F,
        col.by="black", comb.fixed=F, col.diamond.random='black',
col.diamond.lines.random='black',
        col.square = 'blue', col.square.lines = 'blue', print.byvar = F,
test.subgroup.random = F, fontsize = 8, spacing = 0.8)

metainf(cdk_OS, pooled = 'random')
forest(metainf(cdk_OS, pooled = 'random'), layout = 'revman5',
        col.square = 'blue', col.square.lines = 'blue')
```

Figure S1. Visual summary of ROBINS-I for survival outcomes

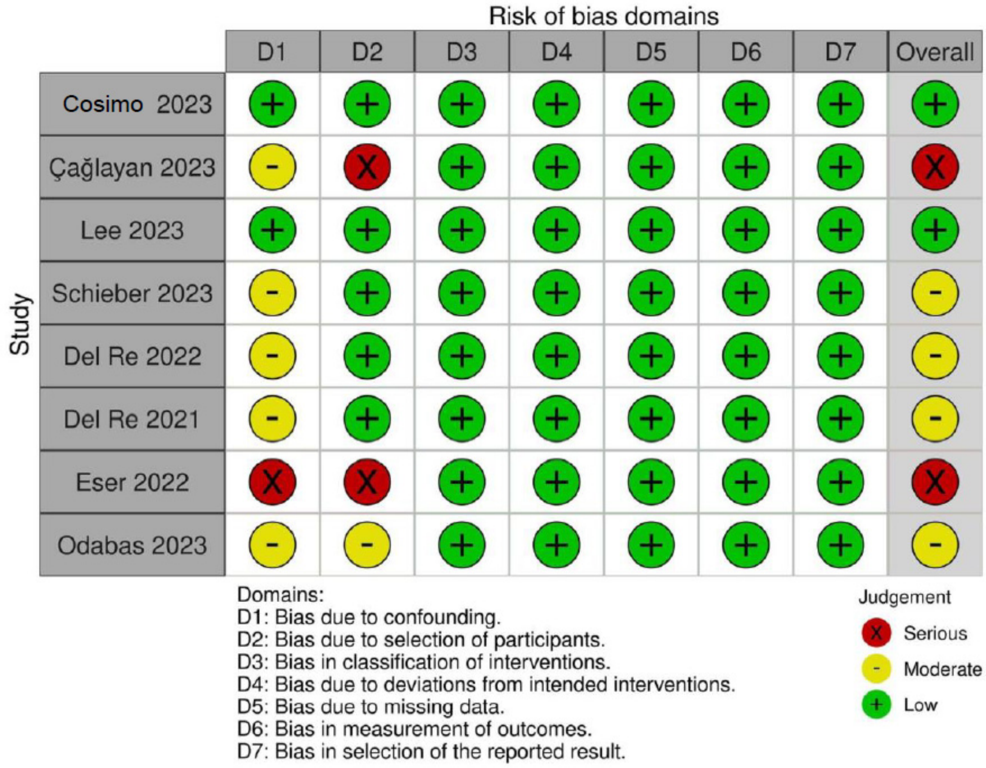

Reference: Cosimo 2023 [30], Çağlayan 2023 [17], Lee 2023 [29], Schieber 2023 [19], Del Re 2022 [28], Del Re 2021 [16], Eser 2022 [20], Odabas 2023 [18]

Figure S2. Leave-one-out analysis of comparative overall survival and progression-free survival between PPI users and non-users in HR+/HER2- metastatic breast cancer patients taking Palbociclib.

Overall Survival

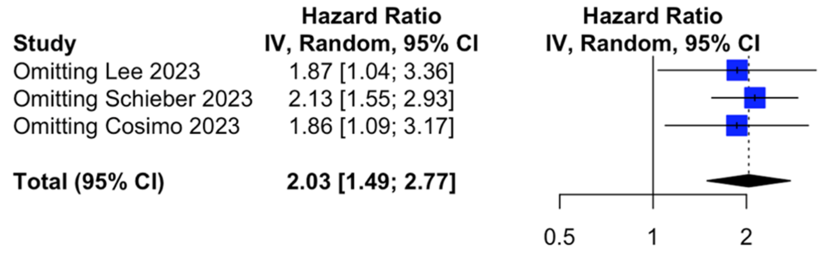

Progression-Free Survival

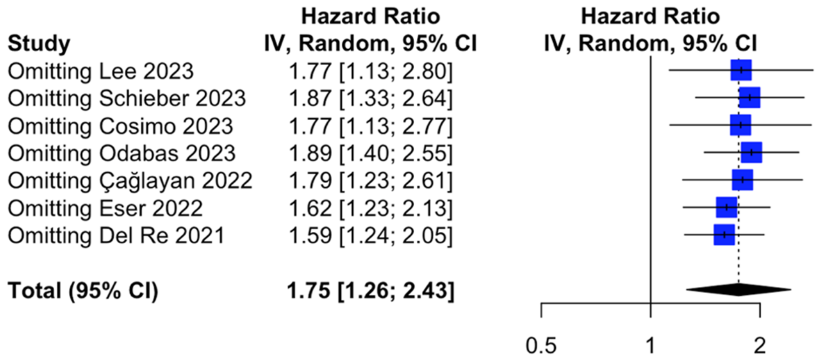

Detail of leave-one-out analysis:

Influential analysis (Random effects model) of overall survival

	HR [95%-CI]	p-value	tau^2	tau	I^2
Omitting Lee 2023	1.8668 [1.0382; 3.3566]	0.0370	0.0559	0.2364	19.3%
Omitting Schieber 2023	2.1287 [1.5470; 2.9292]	< 0.0001	0.0000	0.0000	0.0%
Omitting Cosimo 2023	1.8621 [1.0944; 3.1685]	0.0219	0.0368	0.1917	13.7%
Pooled estimate	2.0347 [1.4933; 2.7724]	< 0.0001	0.0000	0.0013	0.0%

Influential analysis (Random effects model) of progression-free survival

Omitting Lee 2023	1.7744 [1.1257; 2.7970]	0.0135	0.2081	0.4562	65.8%
Omitting Schieber 2023	1.8737 [1.3299; 2.6400]	0.0003	0.1061	0.3257	59.4%
Omitting Cosimo 2023	1.7680 [1.1303; 2.7654]	0.0125	0.2026	0.4501	65.8%
Omitting Odabas 2023	1.8943 [1.4048; 2.5545]	< 0.0001	0.0629	0.2508	53.3%
Omitting Çağlayan 2022	1.7898 [1.2255; 2.6140]	0.0026	0.1501	0.3874	65.5%
Omitting Eser 2022	1.6175 [1.2293; 2.1283]	0.0006	0.0564	0.2375	47.9%
Omitting Del Re 2021	1.5945 [1.2404; 2.0497]	0.0003	0.0343	0.1851	52.8%
Pooled estimate	1.7461 [1.2566; 2.4263]	0.0009	0.1124	0.3353	59.0%

Details on meta-analytical method:
- Inverse variance method. - Restricted maximum-likelihood estimator for tau^2

Reference: Lee 2023 [29], Schieber 2023 [19], Cosimo 2023 [30], Odabas 2023 [18], Çağlayan 2022 [17], Eser 2022 [20], Del Re 2021 [16].

Figure S3. Forest plot of adjusted survival outcomes between PPI users and nonusers in patients taking Palbociclib

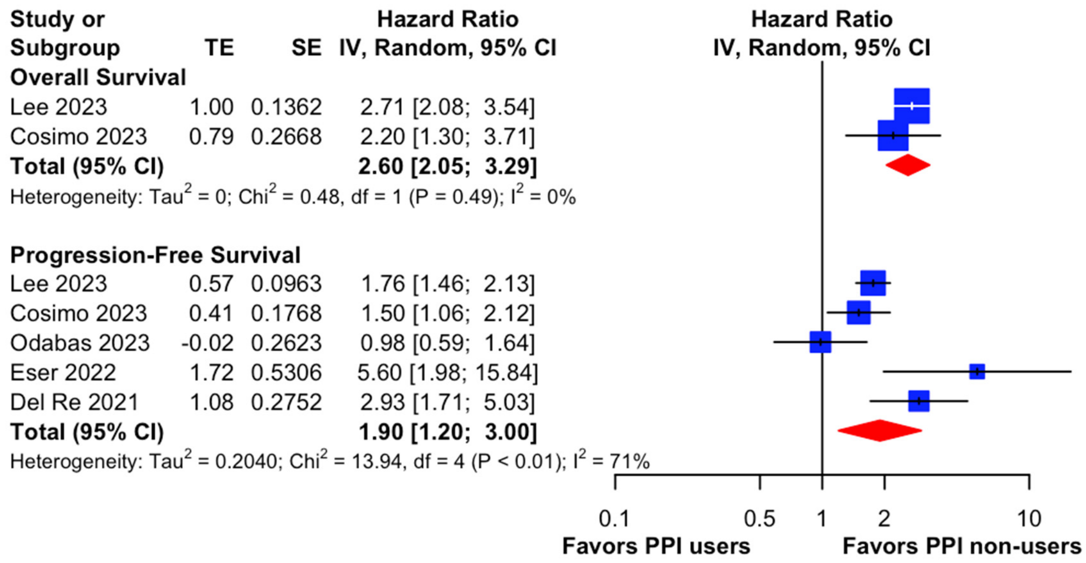

References: Lee 2023 [29], Cosimo 2023 [30],Odabas 2023 [18], Eser 2022 [20], Del Re 2021 [16].

Figure S4. Forest plot of unadjusted survival outcomes between PPI users and nonusers in patients taking Palbociclib after the exclusion of studies subject to serious risk of bias

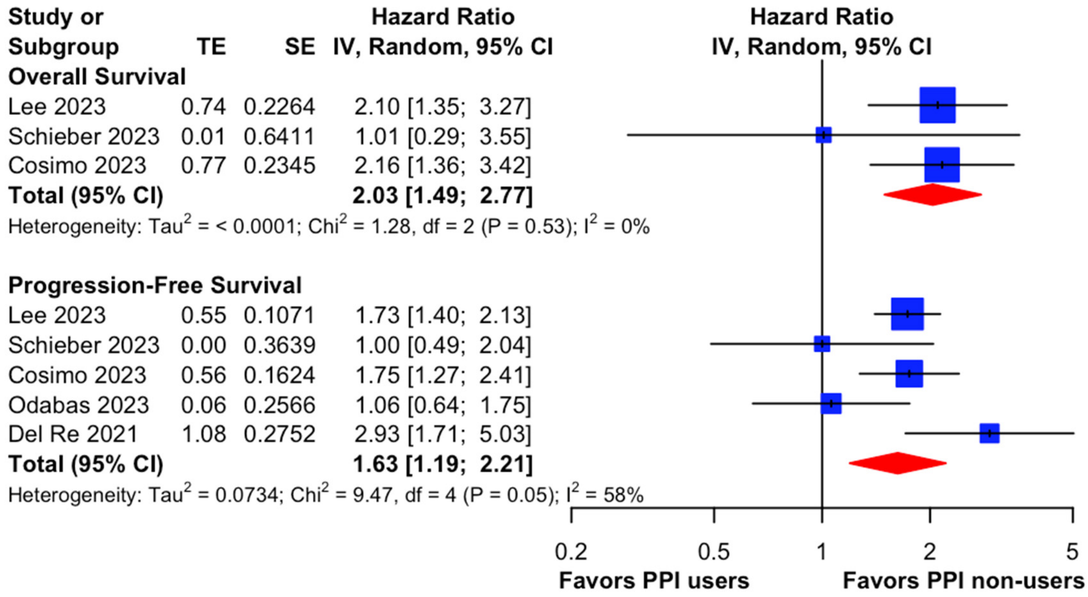

Reference: Lee 2023 [29], Schieber 2023 [19], Cosimo 2023 [30], Odabas 2023 [18], Del Re 2021 [16].

Figure S5. Leave-one-out analysis of comparative progression-free survival between PPI users and non-users in HR+/HER2- metastatic breast cancer patients taking Ribociclib.

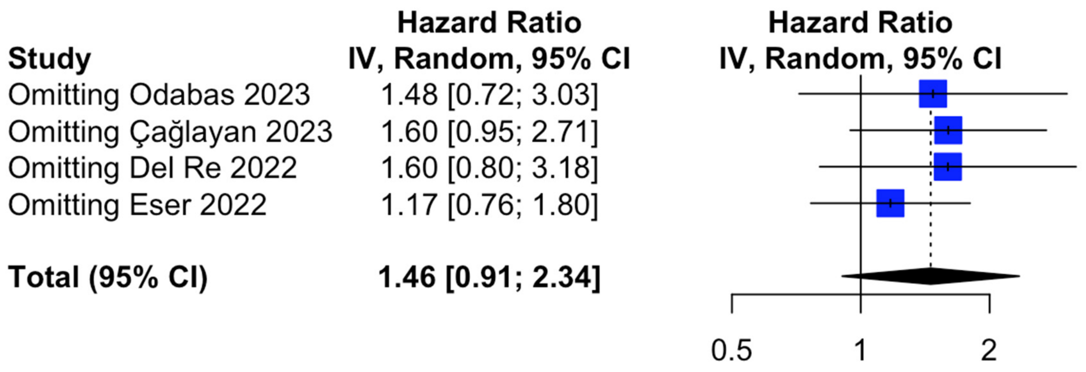

Detail of leave-one-out analysis:

Influential analysis (Random effects model) of progression-free survival

	HR [95%-CI	p-value]	tau^2	tau	I^2
Omitting Odabas 2023	1.4753 [0.7178; 3.0321]	0.2901	0.2275	0.4769	56.8%
Omitting Çağlayan 2023	1.6015 [0.9452; 2.7136]	0.0801	0.0938	0.3062	43.6%
Omitting Del Re 2022	1.5978 [0.8020; 3.1835]	0.1827	0.1752	0.4186	47.8%
Omitting Eser 2022	1.1730 [0.7643; 1.8004]	0.4653	0.0000	0.0000	0.0%
Pooled estimate	1.4567 [0.9051; 2.3445]	0.1213	0.0798	0.2824	35.9%

Details on meta-analytical method:

- Inverse variance method
- Restricted maximum-likelihood estimator for tau^2

References: Odabas 2023 [18], Çağlayan 2022 [17], Del Re 2022 [28], Eser 2022 [20].

Figure S6. Leave-one-out analysis of risk ratio of CDKi dose reduction due to drug toxicity between PPI users and non-users in HR+/HER2- metastatic breast cancer patients taking Palbociclib and Robociclib.

Palbociclib

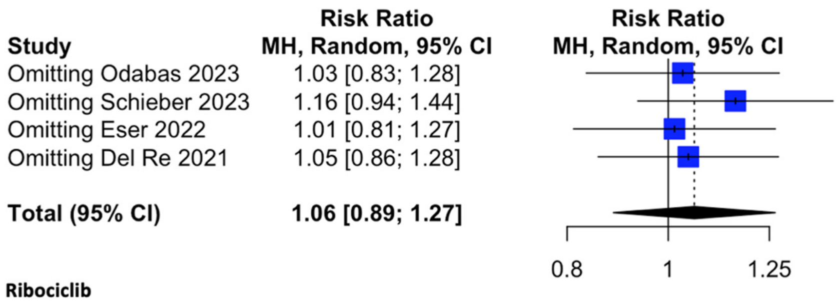

Ribociclib

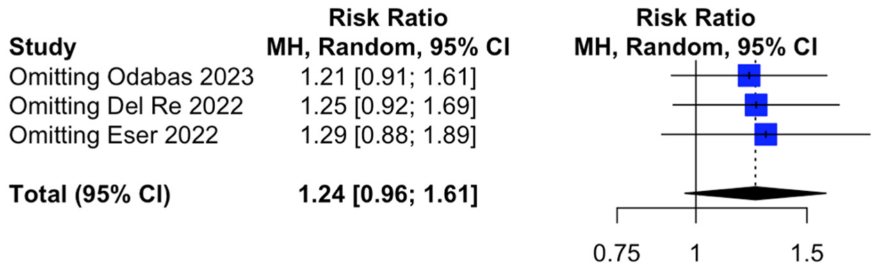

Detail of leave-one-out analysis:

Influential analysis (Random effects model) of palbociclib

	RR [95%-CI].	p-value	tau^2	tau	I^2
Omitting Odabas 2023	1.0326 [0.8345; 1.2776]	0.7680	0.0033	0.0575	0.0%
Omitting Schieber 2023	1.1597 [0.9351; 1.4384]	0.1773	0.0000	0.0000	0.0%
Omitting Eser 2022	1.0139 [0.8115; 1.2666]	0.9036	0.0010	0.0308	0.0%
Omitting Del Re 2021	1.0454 [0.8569; 1.2753]	0.6619	0.0021	0.0458	1.4%
Pooled estimate	1.0593 [0.8861; 1.2663]	0.5270	0.0000	0.0000	0.0%

Influential analysis (Random effects model) of ribociclib

Omitting Odabas 2023	1.2144 [0.9145; 1.6128]	0.1795	0.0000	0.0000	0.0%
Omitting Del Re 2022	1.2461 [0.9203; 1.6872]	0.1547	0.0000	0.0000	0.0%
Omitting Eser 2022	1.2908 [0.8829; 1.8872]	0.1878	0.0000	0.0000	0.0%
Pooled estimate	1.2430 [0.9611; 1.6075]	0.0973	0.0000	0.0000	0.0%

Details on meta-analytical method:

- Inverse variance method
- Restricted maximum-likelihood estimator for tau^2

References: Odabas 2023 [18], Schieber 2023 [19], Eser 2022 [20], Del Re 2021 [16], Del Re 2022 [28].
